# Structural Mechanics Calculations of SiC/Mo-Re Composites with Improved High Temperature Creep Properties

**DOI:** 10.3390/ma18153459

**Published:** 2025-07-23

**Authors:** Ke Li, Egor Kashkarov, Hailiang Ma, Ping Fan, Qiaoli Zhang, Andrey Lider, Daqing Yuan

**Affiliations:** 1 Department of Nuclear Physics, China Institute of Atomic Energy, Beijing 102413, China; like@tpu.ru (K.L.); mhl624@ciae.ac.cn (H.M.); fanping@ciae.ac.cn (P.F.); zql@ciae.ac.cn (Q.Z.); 2 School of Nuclear Science and Engineering, National Research Tomsk Polytechnic University, Tomsk 634050, Russia

**Keywords:** laminated composite, finite element simulation, refractory materials, silicon carbide, molybdenum–rhenium alloy

## Abstract

In the present work, we design a laminated composite composed of molybdenum–rhenium alloy and silicon carbide ceramics for use in space reactors as a candidate structural material with neutron spectral shift properties. The influence of the internal microstructure on the mechanical properties is investigated by finite element simulation based on scale separation. The results of the study showed that the incorporation of gradient transition layers between the metallic and ceramic phases effectively mitigates thermally induced local stresses arising from mismatches in coefficients of thermal expansion. By optimizing the composition of the gradient transition layers, the stress distribution within the composite under operating conditions has been adjusted. As a result, the stress experienced by the alloy phase is significantly reduced, potentially extending the high-temperature creep rupture life.

## 1. Introduction

Providing energy from space nuclear reactors is currently the mainstream solution for energy supply for deep space exploration operations and bases on surface of planets [1]. Due to extremely high operating temperatures (exceeding 1000 °C) and pressure (on the order of several MPa), core structural materials must meet stringent performance requirements. These include long-term thermal creep resistance, a low ductile-to-brittle transition temperature (DBTT), good weldability, and resistance to irradiation-induced swelling and embrittlement. Additionally, these materials should possess low density and maintain compatibility with nuclear fuels as well as alkali liquid metal and gas coolants for service lifetimes of up to 10 years [2]. Moreover, a launch abort accident leading to space reactor submersion in water or wet sand may result in subsequent flooding with water. Water acts as a moderator, scattering fast neutrons and reducing their energy. In such a scenario, the neutron spectrum softens significantly toward the thermal range, which increases neutron capture and fission rates while decreasing leakage. This shift in spectrum can raise reactivity—potentially making the reactor supercritical. Thus, it is a prerequisite for the reactors to be approved for launch that they can remain sufficiently subcritical in such situation. The incorporation of spectral shift absorbers (SSAs) can significantly increase absorption cross-sections for thermal versus fast neutrons, thereby compensating for the reactivity increase.

Refractory metals rhenium (Re) and molybdenum (Mo), which have the advantages of a high melting point (Mo—2623 °C; Re—3186 °C) and excellent mechanical properties, are considered important candidates for structural materials of accident tolerant fuel (ATF) system. Metal Mo has excellent thermal conductivity, electrical conductivity, corrosion resistance, low thermal expansion, and high hardness [3,4]. However, pure Mo has some disadvantages, such as high brittleness at room temperature, recrystallization induced embrittlement, low oxidation resistance, and poor machinability and weldability [5]. The incorporation of metal Re can increase the strength of the alloy and the recrystallization temperature [6], improve the plasticity and weldability, and reduce the DBTT. The molybdenum–rhenium (Mo-Re) alloys have good chemical compatibility with various alkali liquid metal coolants and uranium dioxide ${(UO}_{2})$ up to 1800 K [2,7]. Moreover, rhenium is a neutron SSA [8], which has a large cross-section of thermal neutron absorption and a small cross-section of fast neutron absorption. Therefore, Re can protect the reactor from nuclear criticality safety issues in the accident, which meets the design requirement of fast spectrum space nuclear reactors.

The incorporation of Re brings neutron spectral shift properties and better plasticity to the alloy but also results in poor high-temperature creep properties of Mo-Re alloys. In the creep testing at 1600 °C and 25 MPa, the failure time of Mo-Re alloy is about 10 h, and the creep rate increases significantly when the rhenium content increases to 51 wt.% [9]. The fracture type of the alloy changes from ductile to quasi-brittle at temperatures higher than 1400 °C [10].

Silicon carbide (SiC) has excellent high-temperature creep resistance. The high-temperature flexural strength of SiC at 1600 °C is basically the same as that at room temperature [11]. SiC does not exhibit high-temperature creep phenomena like metals. It is an important candidate material for structural materials of future nuclear reactors. In addition, SiC has an elastic modulus of up to 450 GPa; correspondingly, the elastic modulus of Mo-Re alloy is not higher than 350 GPa (Mo-43Re alloy) [12]. However, the high brittleness and low air tightness of SiC restrict its application in reactor structural materials.

Both SiC and Mo-Re alloy have excellent properties, such as irradiation resistance, high temperature, and corrosion resistance. A wide range of SiC-based composites have been developed using various processing techniques—including chemical vapor infiltration (CVI) [13], polymer infiltration and pyrolysis (PIP) [14], and reactive melt infiltration (RMI) [15]—to achieve controlled fiber architectures and graded microstructures. In previous works, we have demonstrated that laminated SiC_f_/SiC composites fabricated from layered preceramic paper by spark plasma sintering (SPS) have demonstrated significant advantages, enabling precise control over layer composition and microstructure in each lamella. This approach allows tailored reinforcement architectures, leading to high strength, quasi-ductile behavior, and enhanced damage tolerance suitable for nuclear reactor structural materials [16,17,18]. If SiC and Mo-Re are made into laminated composites by using this process, when stress is applied along the direction of layers, the composites have a higher elastic modulus than the Mo-Re alloy, resulting in smaller strains. The stress on the Mo-Re alloy phase of composites is reduced. In other words, the SiC phase bears more stress and reduces the stress borne by the Mo-Re phase. This reduces its high-temperature creep rate, thereby increasing its creep rupture life.

At present, there have been reports on the fabrication of ceramic/metal composite materials by using Mo and SiC [19], achieving a heterogeneous interface and the chemical compatibility of Mo and SiC. Due to similar chemical properties of Re and Mo [20,21], it is expected that the incorporation of Re cannot hinder the fabrication of SiC/Mo-Re composites. In summary, the composites prepared by combining the advantages of Mo-Re and SiC are expected to obtain excellent neutron spectrum shift performance and high temperature creep resistance, which have advantages for potential applications as ATF structural materials of space nuclear reactors (such as cladding tubes, core heat pipes, etc.).

To verify the rationality of the idea, structural mechanics simulation and analysis of the designed SiC/Mo-Re laminated composites are carried out by finite element simulation (FES) approaches. The mechanical behavior of the composite material under space nuclear reactor operating conditions (temperature—~1600 °C and radial stress—5 MPa) is predicted, and the distribution of Mo-Re phase corresponding stress and interface shear stress under different structural designs are calculated. The stress experienced by the Mo-Re phase and the corresponding interface strength requirements when stress acts in the direction of the composite layer are evaluated to optimize the structural design.

## 2. Methodology

### 2.1. Design of Molybdenum-Based Silicon Carbide Composites

As far as we are aware, there exists no published work combining SiC and Mo–Re alloy into laminated composites. We have designed SiC-reinforced Mo-based composites with a laminated structure to obtain a high-temperature creep-resistant structural material with neutron spectrum shift performance.

**Figure 1:**
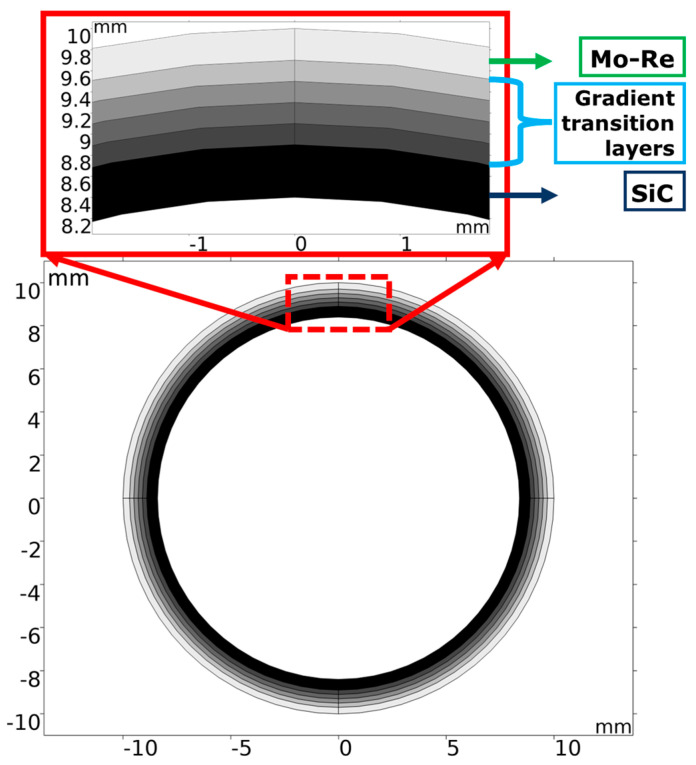
Design schematic diagram of the SiC/Mo-Re composites.

The design schematic diagram of the novel SiC/Mo-Re laminated composites is shown in the Figure 1. The composites consist of the SiC layers, the Mo-Re alloy layers, and the gradient transition layers between the two formers. The thickness of layers in the figure is for illustration only, the specific values of various phases are optimized according to requirements. Fuel cladding materials are subjected to high temperatures and high stresses under operating conditions. SiC has a higher elastic modulus than Mo-Re alloy, so the reasonable incorporation of SiC ceramic layer parallel to the stress direction can reduce the internal stress loaded on the Mo-Re alloy phase under operating conditions, thereby extending its creep life. However, due to the significant difference in the coefficients of thermal expansion (CTEs) (SiC—4~$5\times 10^{-6}/K$; Mo-Re—6~$7\times 10^{-6}/K$) and the incompatibility of heterogeneity between the ceramic and alloy phase, the gradient transition layers, where the fraction of one phase decreases layer by layer and that of the other phase increases layer by layer, are incorporated to play a critical role. This reduces the local thermal stresses inside the laminated composites at various temperatures during fabrication, placement, and exploitation; enhances interfacial bonding between ceramic and metal phases; and improves heterogeneous interface compatibility, while critically contributing to the overall mechanical properties of the composites.

For nuclear reactor structural materials to fulfill their design intent, predicting the maximum stress tolerances without creep failure during the operational lifespan is a fundamental requirement. According to the Monkman–Grant relationship [22,23], the creep rupture life t of an alloy varies inversely with the secondary creep rate $\dot{\varepsilon }$, as follows:

(1) $$t=\frac{M}{\dot{\varepsilon }}$$

where, for a given alloy, the coefficient M can remain constant over broad ranges of stress and temperature. The rate of strain is determined by the following equation [24]:

(2) $$\dot{\varepsilon }=\frac{d\varepsilon }{dt}=A\sigma^{n}e^{-\frac{Q}{RT}}$$

where ε is creep strain; A is constant; n is the stress exponent determined from the stress–strain curve, which depends on the creep mechanism (in this work, the value should be taken as 4~5, since dislocation processes determine the creep properties of pure metals at high stresses) [25]; σ is applied stress; R is gas constant; T is temperature; and Q is activation energy determined from the natural/log of creep rate plotted against the reciprocal of temperature. So obviously, when the temperature is constant, we can obtain a dependence of the creep rupture life t on applied stress by the following derivation process:

(3) $$t\sigma^{n}\propto constant\;\mathrm{lg}t\sigma^{n}\propto constant\;n\mathrm{lg}\sigma +\mathrm{lg}t=C$$

where C is a constant.

According to experimental data in reference [9], the following relationship at 1600 °C for Mo-14Re alloy can be approximated as follows:

(4) 2.37lgσ+lgt=7.62

where n=2.37, C=7.62. When $t>10^{5}$ h, we obtained σ<12.75 MPa. In other words, to ensure that the creep rupture life of Mo-14Re reaches $10^{5}$ h (>10 years) at 1600 °C, the stress loaded should not exceed 12.75 MPa. Therefore, the design should try to reduce the stress of the Mo-Re alloy phase in the SiC/Mo-Re composites under the operating conditions (temperature, pressure, etc.).

In practical operating conditions, the gaseous fission products exert pressure on the cladding [26], and the irradiation-induced swelling of the fuel pellets leads to pellet–cladding mechanical interaction (PCMI), which also causes mechanical stresses on the cladding [27]. For this reason, we set the value of cladding internal pressure to 5 MPa, which is higher than the radial stress experienced by the cladding under the operating conditions [27,28], to reflect the influence of this part. In addition, the axial temperature distribution in claddings can also induce thermal stresses, but axial temperature gradients are generally orders of magnitude smaller than radial gradients in steady-state reactor operations. Consequently, for the scope of this study, axial thermal stress is not explicitly included. However, potential relevance under non-uniform axial heating scenarios could be addressed in future work.

### 2.2. Calculations on Structural Mechanics by FES

As described above, the design of composites focuses on reducing the stress within the Mo-Re alloy phase under operating conditions to extend the failure lifetime to an acceptable range (assumed to be $10^{5}$ h in this work). To analyze the distribution of internal stress in the designed composites and optimize the design, the finite element simulation (FES) method was used to conduct numerical calculations of structural mechanics for the composites. The calculation of the stress distribution inside SiC/Mo-Re laminated composites under operating conditions is carried out taking into account the pressure of gaseous fission products and the pellet–cladding mechanical interaction, corresponding to excess operating parameters.

For the simulation of composites, approaches that are usually applied include a micromechanics-based approach and a equivalent homogeneous material (EHM)-based approach, each of them having advantages and disadvantages [29,30]. The micromechanical based approach describes the material behavior locally, and thereby, it is possible to study regions related to gradient transition layers with different phase proportions, including complex interfaces [31]. However, the study on mechanical behavior locally requires the mesh to be set extremely finely compared to the EHM approach, which greatly slows down the calculation efficiency [32]. Directly incorporating the transition layers with the micrometer-scale microstructure into the millimeter-scale cladding tube model is not considered. The EHM approach has obvious advantages in simulation time, but it is not able to predict local effects and is not able to be directly used for gradient transition layers.

**Figure 2:**
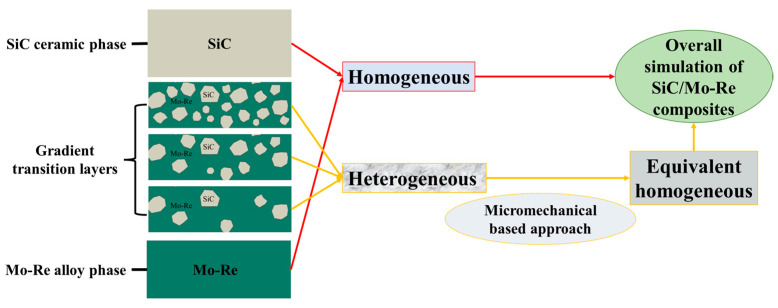
Schematic diagram of the FE simulation for the SiC/Mo-Re composites.

As shown in Figure 2, in this study, the advantages of the above two approaches are taken, and the corresponding approaches are used in different regions in composites to predict structural mechanics behavior with high efficiency; therefore, scale separation is necessary. On the FES of the SiC/Mo-Re composites, the material is considered in the following parts—homogeneous layer and gradient transition layer. An EHM-based approach is used for various heterogeneous layers in the gradient transition layers, the parameters of which are given by a micromechanics-based approach.

The present work adopts a 2D finite element (FE) model of a cladding tube based on SiC/Mo-Re composites (Figure 1), which has an outer diameter of 10 mm and consists of a SiC ceramic layer, a Mo-Re alloy layer, and a series of translation layers. These materials serve as fuel cladding in lithium-cooled fast reactors, where operational temperatures can reach ~1600 °C. The composites are intended to be prepared by sintering at approximately 2000 °C; the dominant residual thermal stress developed during cooling is released as the temperature decreases to around 1200 °C, driven by mechanisms, such as grain-boundary diffusion, creep, and microplastic relaxation [33,34]. Cooling further to room temperature does induce renewed thermally induced stress due to differential contraction, but upon increasing back to ~1200 °C, this secondary stress is largely relieved again. Therefore, 1200 °C is chosen as the reference (zero internal thermal stress) temperature for the simulations.

In this work, the metallic (Mo-Re) layer and the ceramic (SiC) layer are considered as homogeneous phases. In the calculations, some temperature-sensitive parameters (such as the heat capacity of Mo-Re, the thermal conductivity of both phases) within the studied temperature range are represented by using interpolations.

The gradient transition layers of the SiC/Mo-Re laminated composites are composed of various proportions of alloy phase and ceramic phase. Obviously, the mechanical properties of such layers may rely on the geometric characteristics of internal microstructure (such as size and volume fraction of SiC particles). Therefore, it is required to study the influence of the geometric parameters of SiC particles on the intrinsic material parameters of such a region.

For this case, the properties for each heterogeneous layer are given by micromechanics analysis, then various gradient transition layers of the composite are modeled by an EHM approach. The corresponding properties of gradient transition layers with various parameters are calculated independently by using a micromechanics-based approach, which is performed for six load cases and material data for a homogenized material is created [35,36]. Specifically, the mechanical behaviors of a heterogeneous layer are described by using the representative volume elements (RVEs) in the FES approaches, i.e., RVEs obtain effective properties that represent the properties of heterogeneous gradient transition layers on the macroscopic scale [37,38]. Therefore, in order to numerically model gradient transition layers containing various equivalents of SiC particles, it is important to generate RVEs with various fractions of SiC particles with corresponding randomly distributed shapes, sizes, and positions.

The widely adopted simplified construction of particles (such as setting particles as linear elastomers, arranging particle shape as sphere, assigning uniform particle size and distribution, etc.) in FES inevitably deteriorate the accuracy of prediction results [39]. Therefore, in this work, FE models of gradient layers were established with SiC particles, which have randomly distributed arbitrary polyhedral shapes, with a specified size.

**Figure 3:**
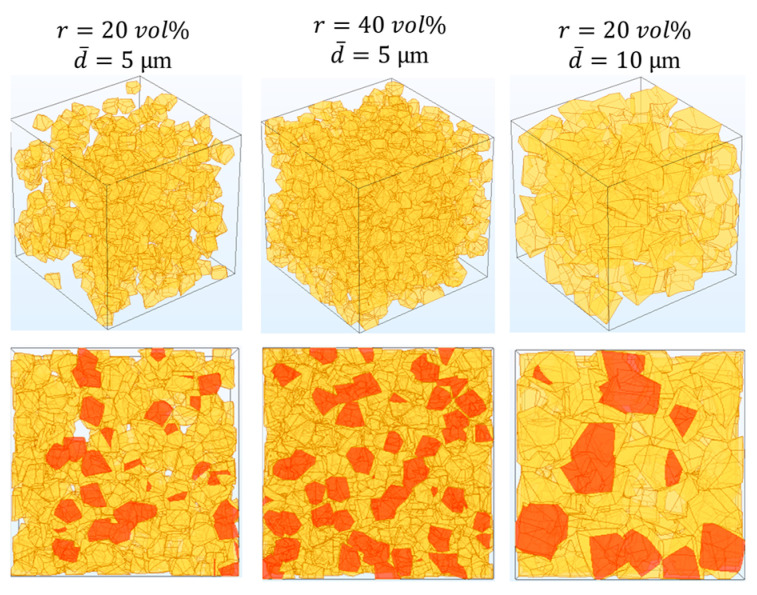
Microstructure of the gradient transition layers of the SiC/Mo-Re composites with various parameters (r, $\bar{d}$—volume fractions, mean particle size of SiC particles; the yellow regions correspond to SiC particles, and the red regions in the 2D images represent SiC particles intersected by the cross-sectional plane).

For the gradient transition layers of the SiC/Mo-Re composites, the heterogeneous material consists with a volume fraction of 20 vol%, 40 vol%, 60 vol%, and 80 vol% randomly distributed polygon-shaped SiC particles and the RVEs are set up as cubes with side lengths of 50 μm. In addition, layers with different particle sizes were also created to analyze the effect of the particle size on final properties. The SiC particle sizes follow a log-normal distribution with σ = 0.1 in the log-domain. Mechanically milled ceramic powders commonly exhibit narrow, right-skewed particle size distributions that conform to log-normal statistics [40]. The Weibull distribution is also frequently applied to describe milled powder size spectra and could be explored in future work to assess the sensitivity of properties to size distribution. Figure 3 shows the microstructures of the gradient transition layers.

**Figure 4:**
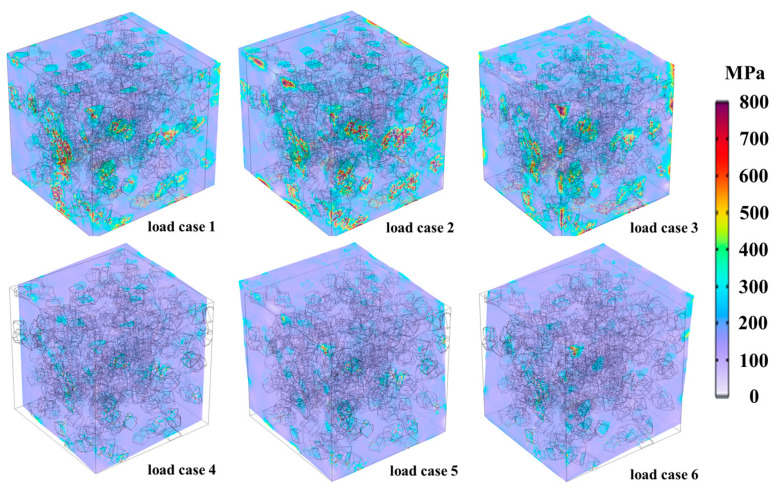
Von Mises stress distribution together with the deformation in a RVE (with a volume fraction of 20 vol% and a mean particle size of 5 μm of SiC particles) for six different load cases.

Periodic boundary conditions are applied to the RVEs. Six independent load cases are used to determine the elastic modulus tensor of the equivalent homogenized transition layer (unit average strain of 0.1% applied in each case). Finally, the elastic modulus and Poisson’s ratio for each layer within the full gradient transition structure are obtained. Figure 4 shows the stress distribution of Von Mises under six different load cases and the deformation in an RVE.

In the micromechanics analysis, based on the assumption of linear elasticity, the stress tensor components can express the strain tensor components of transition layer linearly according to Hooke’s law:

(5) $$\left\{\sigma \right\}=\left[C\right]\left\{\varepsilon \right\}$$

where $\left\{\varepsilon \right\}$ and $\left\{\sigma \right\}$ are strain and stress, respectively; $\left[C\right]$ is the elastic modulus tensor. In this work, we have the following forms:

(6) $$\left[\begin{array}{c} \sigma_{x}\\ \sigma_{y}\\ \sigma_{z}\\ \tau_{xy}\\ \tau_{xz}\\ \tau_{yz} \end{array}\right]=\left[\begin{array}{c} C_{11}\\ C_{21}\\ C_{31}\\ C_{41}\\ C_{51}\\ C_{61} \end{array}\begin{array}{c} C_{12}\\ C_{22}\\ C_{32}\\ C_{42}\\ C_{52}\\ C_{62} \end{array}\begin{array}{c} C_{13}\\ C_{23}\\ C_{33}\\ C_{43}\\ C_{53}\\ C_{63} \end{array}\begin{array}{c} C_{14}\\ C_{24}\\ C_{34}\\ C_{44}\\ C_{54}\\ C_{64} \end{array}\begin{array}{c} C_{15}\\ C_{25}\\ C_{35}\\ C_{45}\\ C_{55}\\ C_{65} \end{array}\begin{array}{c} C_{16}\\ C_{26}\\ C_{36}\\ C_{46}\\ C_{56}\\ C_{66} \end{array}\right]\times \left[\begin{array}{c} \varepsilon_{x}\\ \varepsilon_{y}\\ \varepsilon_{z}\\ \gamma_{xy}\\ \gamma_{xz}\\ \gamma_{yz} \end{array}\right]$$

where ε and γ are the normal and shear strain tensor components, respectively; σ and τ are the normal stress tensor components and the shear stress tensor components, respectively; and $C_{ij}$ is the elastic modulus. Since the SiC particles are set to be randomly distributed, the transition layer is considered isotropic at the mesoscopic scale. Therefore, the elastic modulus tensor of the transition layer can be rewritten as follows [41]:

(7) $$\left[C\right]=\left[\begin{array}{c} C_{11}\\ C_{12}\\ C_{12}\\ 0\\ 0\\ 0 \end{array}\begin{array}{c} C_{12}\\ C_{11}\\ C_{12}\\ 0\\ 0\\ 0 \end{array}\begin{array}{c} C_{12}\\ C_{12}\\ C_{11}\\ 0\\ 0\\ 0 \end{array}\begin{array}{c} 0\\ 0\\ 0\\ \frac{C_{11}-C_{12}}{2}\\ 0\\ 0 \end{array}\begin{array}{c} 0\\ 0\\ 0\\ 0\\ \frac{C_{11}-C_{12}}{2}\\ 0 \end{array}\begin{array}{c} 0\\ 0\\ 0\\ 0\\ 0\\ \frac{C_{11}-C_{12}}{2} \end{array}\right]\;=\frac{E}{\left(1+\nu )(1-2\nu \right)}\left[\begin{array}{c} 1-\nu \\ \nu \\ \nu \\ 0\\ 0\\ 0 \end{array}\begin{array}{c} \nu \\ 1-\nu \\ \nu \\ 0\\ 0\\ 0 \end{array}\begin{array}{c} \nu \\ \nu \\ 1-\nu \\ 0\\ 0\\ 0 \end{array}\begin{array}{c} 0\\ 0\\ 0\\ \frac{1-2\nu }{2}\\ 0\\ 0 \end{array}\begin{array}{c} 0\\ 0\\ 0\\ 0\\ \frac{1-2\nu }{2}\\ 0 \end{array}\begin{array}{c} 0\\ 0\\ 0\\ 0\\ 0\\ \frac{1-2\nu }{2} \end{array}\right]$$

where E and ν are the Young’s modulus and the Poisson’s ratio, respectively. That is, the constitutive equation has only 2 independent variables [42], as follows:

(8) $$E=\frac{\left(C_{11}-C_{12})(C_{11}+2C_{12}\right)}{C_{11}+C_{12}}$$

(9) $$\nu =\frac{C_{12}}{C_{11}+C_{12}}$$

The elastic modulus and Poisson’s ratio calculated due to different conditions are used as material properties for the corresponding transition layers. In order to reduce the adverse impact of anisotropy caused by randomly distributed particles on the results, five RVE models are independently established for various parameters, and finally, the average elastic modulus tensors are obtained.

## 3. Results and Discussion

### 3.1. Intrinsic Properties of Gradient Transition Layers

The volume average stress and strain are obtained by applying six independent loads to the RVEs. As an example, the simulation result of the elastic modulus tensor of the transition layer with a fraction of 40 vol% and mean SiC particle size of 5 μm are as follows:

(10) $$\left[C\right]=\left[\begin{array}{c} 4.95\times 10^{5}\\ 2.82\times 10^{5}\\ 2.81\times 10^{5}\\ 0\\ 0\\ 0 \end{array}\begin{array}{c} 2.82\times 10^{5}\\ 4.98\times 10^{5}\\ 2.82\times 10^{5}\\ 0\\ 0\\ 0 \end{array}\begin{array}{c} 2.81\times 10^{5}\\ 2.82\times 10^{5}\\ 4.97\times 10^{5}\\ 0\\ 0\\ 0 \end{array}\begin{array}{c} 0\\ 0\\ 0\\ 1.07\times 10^{5}\\ 0\\ 0 \end{array}\begin{array}{c} 0\\ 0\\ 0\\ 0\\ 1.07\times 10^{5}\\ 0 \end{array}\begin{array}{c} 0\\ 0\\ 0\\ 0\\ 0\\ 1.07\times 10^{5} \end{array}\right]$$

**Figure 5:**
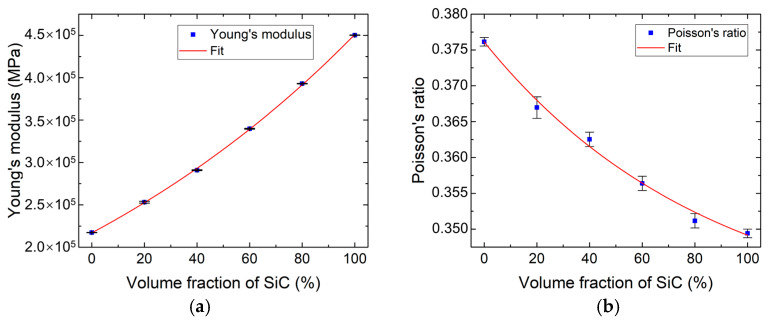
Young’s modulus (**a**) and Poisson’s ratio (**b**) versus the volume fraction of SiC particles in gradient transition layers.

In Equation (10), the calculated anisotropic terms in the elastic modulus tensor matrix are at least three orders of magnitude smaller than the other components. This results from the random distribution of SiC particles within the RVE matrix. Consequently, these negligible terms are set to zero in subsequent calculations. The elastic modulus E and Poisson’s ratio ν calculated by Formulas (8) and (9) are $2.91\times 10^{5}\;MPa$ and 0.36, respectively. The final calculated constants of mechanical properties are shown in Figure 5. The data corresponding to the curves is fitted by a nonlinear regression fitting method based on the least squares method (LSM) to obtain analytical models for mechanical properties, including Young’s modulus E and Poisson’s ratio ν, with versus volume fraction of SiC particles x in gradient transition layers. The fitting function is defined as follows:

(11) $$y=Ae^{-\frac{x}{t}}+y_{0}$$

where y represents the above series of physical quantities; and A, t, and $y_{0}$ represent the fitting coefficients. In this way, the functions of various properties with respect to volume fraction of SiC particles x are obtained.

The influence of the SiC volume fraction on the elastic properties of gradient transition layers were analyzed. As the volume fraction of SiC particles increases, both Young’s modulus and Poisson’s ratio exhibit nonlinear variations. Young’s modulus of the gradient transition layer progressively increases from values approaching pure Mo-Re to those characteristics of SiC, with the rate of increase accelerating at higher volume fractions. Conversely, Poisson’s ratio decreases from Mo-Re values toward SiC values, though the rate of decrease diminishes with increasing SiC content. The SiC particles have a higher modulus but lower Poisson’s ratio than the Mo-Re alloy matrix in transition layers, so the increase in the volume fraction of SiC particles has a positive contribution to the Young’s modulus but a negative contribution to the Poisson’s ratio of such layers. As the volume fraction of SiC particles increases, the interparticle spacing decreases, leading to more frequent particle interactions. This enhanced interaction facilitates a more efficient load transfer from the matrix to the reinforcing particles, thereby increasing the composite’s overall stiffness [43,44]. The simulation results have relatively larger errors when the SiC particle fraction is 20 vol.%. The primary reason is the relatively small number of particles within the RVEs in this situation, leading to insufficient statistical homogeneity, which adversely affects the isotropic properties of RVEs.

**Figure 6:**
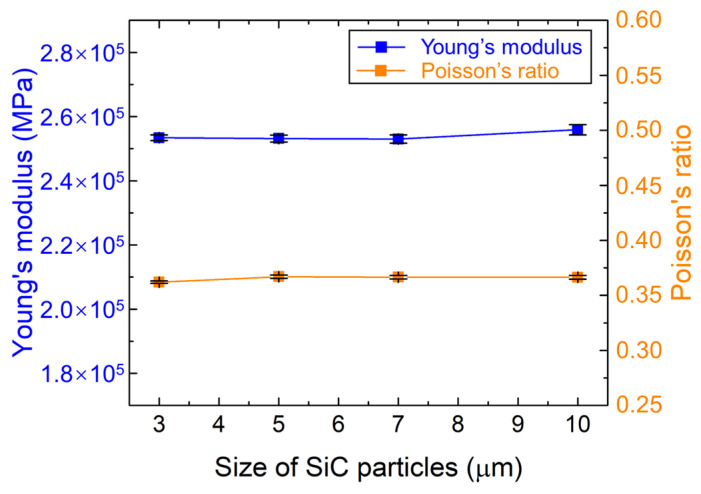
Young’s modulus and Poisson’s ratio versus size of SiC particles in gradient transition layers (20 vol.% of SiC).

Figure 6 shows that Young’s modulus and Poisson’s ratio of the gradient transition layers are not very sensitive to differences in the size of the SiC particle. This result is consistent with the finding of Cho et al., that particle sizes at the micron scale have little influence on the Young’s modulus of the composites [45]. Within the elastic regime, neglecting the effects of interfacial strength, cracking, and other nonlinear factors, the influence of particle size on the elastic modulus is primarily attributed to the uniformity of particle distribution [46]. In this study, SiC particles are randomly and uniformly distributed within the RVE, and the RVE size is significantly larger than the particle size. Moreover, multiple simulations were conducted for each set of parameters to ensure statistical homogeneity. Consequently, the effect of particle size on the uniformity of particle distribution within RVEs can be neglected, leading to negligible variations in elastic modulus and Poisson’s ratio across different particle sizes.

**Figure 7:**
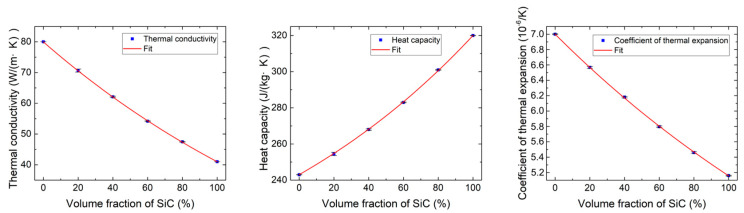
Thermal conductivity, heat capacity, and coefficient of thermal expansion versus volume fraction of SiC particles in gradient transition layers.

Figure 7 illustrates the influence of SiC volume fraction on thermal conductivity, heat capacity, and the coefficient of thermal expansion. The data is fitted by a nonlinear regression fitting method (Formula (11)) based on the LSM to obtain analytical models for thermal properties, including thermal conductivity k, heat capacity C, and the coefficient of thermal expansion α, versus the volume fraction of SiC particles x in gradient transition layers. With the increase in the volume fraction of SiC particles, these thermal properties of the gradient transition layer increase from a value close to that of Mo-Re to that of SiC. Notably, the thermal properties of the transition layers change basically linearly with the increase in the SiC volume fraction. The CTE of the mixed phase is primarily determined by the intrinsic thermal expansion properties of the constituent metal and ceramic phases. This behavior can be effectively predicted by classical mixing rules [47], so the CTE exhibits an approximately linear relationship with the volume fraction of SiC. Similarly, thermal conductivity and heat capacity both exhibit an approximately linear relationship with the volume fraction of SiC, which aligns with predictions made by mixture rules under the conditions of uniform reinforcement distribution [48]. The observed trend is consistent with the findings reported by Kundalwal et al. [48,49] and Hsu et al. [50], where the variation of the thermal properties with reinforcement content in nano- and microscale composites was found to closely follow predictions made by mixture rules. In contrast, mechanical properties, such as Young’s modulus and Poisson’s ratio, often display more pronounced nonlinear behavior due to complex interactions within the composite microstructure.

### 3.2. Intrinsic Properties of SiC/Mo-Re Laminated Composites

In order to characterize the influence of the gradient transition layers, two forms of composites without transition layers and with transition layers are simulated. Each discrete layer within the transition zone is treated as an EHM. The mechanical properties derived from RVE simulations are assigned to their respective layers, enabling the construction of a complete SiC/Mo-Re composite model. This computational framework is then subjected to various operational loading scenarios. The outer surface temperature of the cladding tube is set to 1600 °C, while the inner is set to 1700 °C to simulate the temperature gradient of real operating conditions. Considering the impact of the fission gas that may be released from the fuel inside the cladding tube during operation, a pressure of 5 MPa is set on the inner surface of the tube.

**Figure 8:**
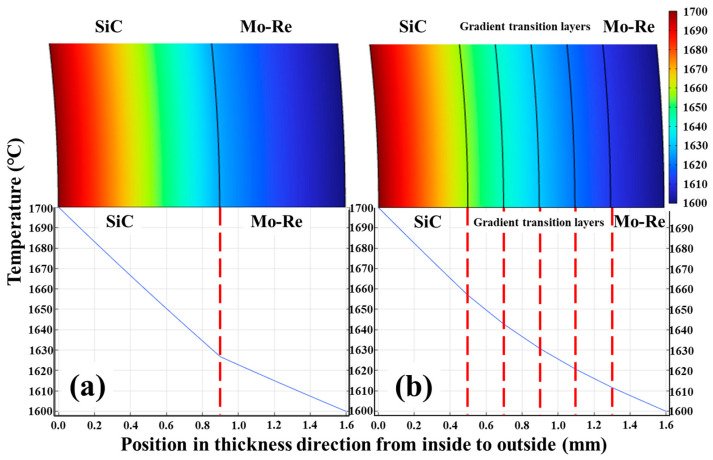
Curves of temperature distribution of the tube without (**a**) and with transition layers (**b**).

The curves of temperature distribution along the radial direction are shown in Figure 8. In conventional metal–ceramic Mo-Re/SiC composites without transition layers, the abrupt discontinuity in thermophysical properties (thermal conductivity and heat capacity) creates significant temperature field distortion at the interface. The incorporation of gradient transition layers eliminates this discontinuity, resulting in a smooth, continuous temperature distribution with more uniform thermal gradients.

**Figure 9:**
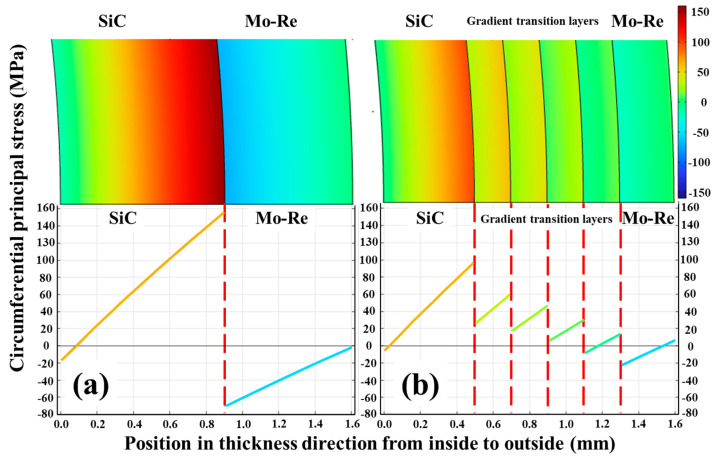
Curves of circumferential stress versus radial thickness of the tube without (**a**) and with transition layers (**b**).

The curves of circumferential principal stress distribution along the radial direction are shown in Figure 9. Obviously, due to the absence of a gradient transition layer, the circumferential stress has a large “jump” at the interface between the metal and ceramic phase. Correspondingly, the incorporation of the gradient transition layers has greatly reduced this “jump”. For the composite without transition layers, the maximum circumferential principal stress in the SiC phase reaches 157.0 MPa, and the Mo-Re phase reaches a compressive stress of 70.9 MPa. While for composite with transition layers, these values are greatly reduced, the circumferential principal stress of SiC phase is a tensile stress not exceeding 98.7 MPa, the stress of Mo-Re phase and transition layers mainly composed of Mo-Re alloy does not exceed a compressive stress of 22.6 MPa.

**Figure 10:**
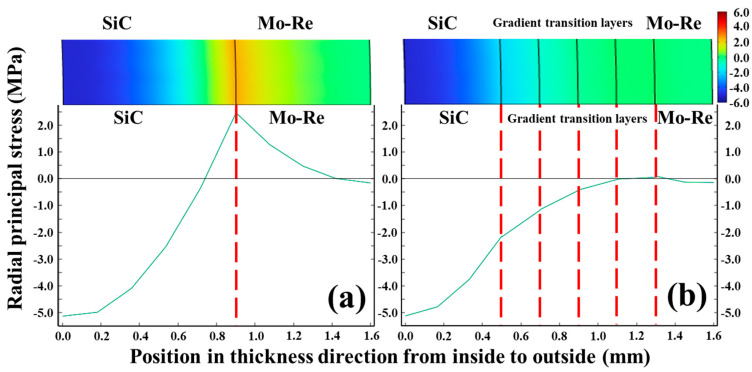
Curves of radial stress versus radial thickness of the tube without (**a**) and with transition layers (**b**).

The curves of radial principal stress distribution along the radial direction are shown in Figure 10. In the composite system without transition layers, the mismatch in thermal conductivity and heat capacity between the ceramic and matrix phases generates a radial principal tensile stress of 2.5 MPa at their interface under operational thermal gradients and mechanical loading. This interfacial tensile stress adversely affects the bonding integrity between phases. The incorporation of the gradient transition layers leads to the disappearance of the tensile stress in the normal direction of the interface. The values of normal stress at the two interfaces close to the Mo-Re phase tend to zero, while those at the other three interfaces appear as compressive stress not exceeding 2.2 MPa. The presence of the transition layers changes the normal stress of the interface under operation conditions, which helps to improve the interface strength of the SiC/Mo-Re composites.

The implementation of graded transition layers not only alleviates detrimental radial tensile stresses but also significantly reduces circumferential stresses of the alloy phase and transition layers mainly composed of alloy, thereby enhancing the overall damage resistance of the composite system under realistic service conditions. In actual applications, compared with radial stresses, circumferential stresses are the critical drivers for damage initiation and crack propagation along the radial direction in cladding structures [51,52]. Therefore, minimizing circumferential stress is of greater significance in preventing crack nucleation and subsequent radial crack growth.

### 3.3. Optimization of Microstructure

The mitigation of stress concentrations is critical for reducing localized failure and enhancing the overall strength of composites. Consequently, design optimization must address the following two key objectives: (1) improving stress distribution patterns and (2) reducing operational stresses in both the Mo-Re phase and Mo-Re-dominated transition layers. Considering practical constraints in composite fabrication, particularly to avoid a high number of gradient transition layers or particles of varying sizes, this study focuses on optimizing the mechanical properties of SiC/Mo-Re composites through the precise regulation of transition layer composition ratios.

Based on the fitting results of the physical properties, including Young’s modulus E, Poisson’s ratio ν, thermal conductivity k, heat capacity C, and the coefficient of thermal expansion α, in Figure 5 and Figure 7, the functions of various properties with respect to the volume fraction of SiC particles x are obtained. That is, x becomes the only parameter to express the various properties of the transition layers. The x of each layer in the transition layers is used as the optimization parameter, and the minimum value of the integral of the circumferential stress along the thickness direction in the Mo-Re phase and the transition layers dominated by Mo-Re (two transition layers adjacent to the Mo-Re phase) is taken as the optimization target.

The parameters and properties of each layer in the optimized gradient transition layers are shown in Table 1. After optimization, the proportion of Mo-Re in each layer has increased, which reduces the component gradient between the Mo Re phase and the transition layers dominated by Mo-Re, thereby reducing the stress.

**Figure 11:**
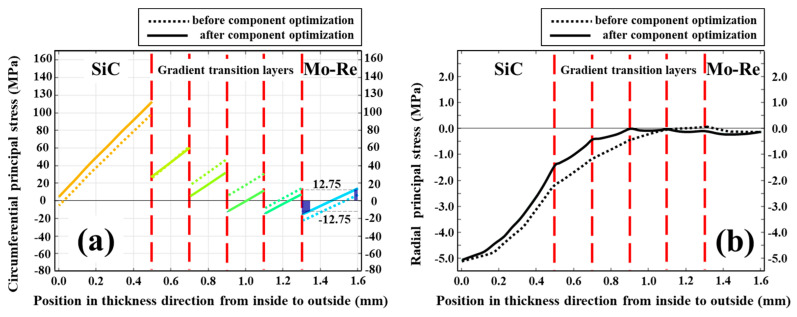
Curves of circumferential (**a**) and radial stress (**b**) versus radial thickness before and after component optimization of transition layers.

The results on circumferential principal stress distribution before and after the component optimization of gradient transition layers are shown in Figure 11a. In the optimized composite, the SiC phase shares more circumferential stress, while the principal stress of the remaining parts has been reduced to varying degrees. In particular, the stress of the Mo-Re phase and the transition layers dominated by Mo-Re are greatly reduced, and the stress value in these area does not exceed 16.0 MPa, which is almost half of that before optimization (29.0 MPa). This value is much lower than the ultimate tensile strength at this temperature [9]. In this case, the corresponding strain of the Mo-Re phase is 0.27%, which is much lower than its elongation to failure [9,53]. The value of maximum stress of the Mo-Re phase is close to the result (12.75 MPa) calculated by Formula (4), and only 20.1% of the Mo-Re phase in the thickness direction exceeds this limit (dark blue part in Figure 11). Even without accounting for high-temperature stress relaxation in the Mo-Re phase or stress redistribution to the SiC phase during Mo-Re creep (which reduces stress in the Mo-Re phase), the creep life of the Mo-Re phase approaches 10^5^; hours.

The results on radial principal stress distribution before and after component optimization of the gradient transition layers are shown in Figure 11b. The values of normal stress at various interfaces in the optimized composite are further greatly reduced. The normal stress values at three (two before optimization) interfaces close to the Mo-Re phase tend to zero. The values of normal stress at the other two interfaces are 0.4 MPa and 1.4 MPa of compressive stress, respectively. Those values are 2.2 MPa and 1.2 MPa before and after optimization, respectively.

RVEs are established for the optimized various layers (volume fraction of SiC particle 67.2 vol%, 44.1 vol%, 23.8 vol%, and 11.5 vol%, respectively) and simulated to verify the fitting results of physical properties. The comparisons between simulation results and the fitting results of the mechanical properties and thermal properties are shown in Table 2 and Table 3, respectively. It can be seen from the tables that the fitting results of each property are consistent with the simulation results of RVEs, and the deviation does not exceed 1%. The optimized component regulation of transition layers can provide a reference for the fabrication of physical specimens, narrowing the scope of trial-and-error during the manufacturing process. Moreover, this simulation-driven optimization strategy can be applied to composite specimens more broadly, providing clear guidance on property-targeted improvements and offering quantitative data to support further enhancements.

## 4. Conclusions

Novel designed SiC/Mo-Re composites were constructed by FES for predicting their mechanical and thermal properties. The effect of the geometric characteristics of internal microstructure as well as of the external stress on structure and mechanical properties of the composites was revealed. The component regulations of the gradient transition layers were optimized to reduce the stress within the composites. The properties of gradient transition layers were predicted by constructing heterogeneous RVEs to provide input properties for the heterogeneous phases of the composite. According to the results, the following conclusions were made:

1. Adding the gradient transition layers between the metallic and ceramic phases reduces the thermally induced local stresses caused by CTE mismatch of various phases inside the composites.
2. The Young’s modulus and Poisson’s ratio of the gradient transition layers are insensitive to differences in the SiC particle size when the shapes, sizes, and positions are randomly distributed.
3. By adjusting the components of various gradient transition layers, the stress distribution in various phases of the composite under operation conditions can be optimized to further reduce the stress in the Mo-Re alloy component.

The incorporation of SiC in laminated SiC/Mo-Re composites reduces the average operational stress in the Mo-Re alloy phase to below 4 MPa, corresponding to an expected high-temperature creep rupture life exceeding 10^4^ h. These results demonstrate the feasibility of the SiC/Mo-Re laminated composite design for meeting operational requirements for application as space reactor structural materials. Further work will focus on the simulation of failure modes of SiC/Mo-Re composites, the fabrication of physical specimens, and the study of their properties for the application in the field of nuclear energy.

## Figures and Tables

**Table 1:** Properties and composition control parameters of various gradient transition layers after optimization.

Layers	Young’s Modulus (GPa)	Poisson’s Ratio	$\mathrm{Thermal}\;\mathrm{Conductivity}\;(\frac{W}{m\cdot K})$	$\mathrm{Heat}\;\mathrm{Capacity}\;(\frac{J}{kg\cdot K})$	$\mathrm{Coefficient}\;\mathrm{of}\;\mathrm{Thermal}\;\mathrm{Expansion}\;(10^{-6}/K)$	Volume Fraction of SiC Particle (vol %)
1	445	0.355	51.73	289.2	5.68	67.2
2	390	0.360	60.41	271.1	6.09	44.1
3	348	0.367	68.90	257.2	6.49	23.8
4	325	0.371	74.47	249.6	6.75	11.5

**Table 2:** Comparison of fitting and simulation results of mechanical properties of optimized transition layers.

Layers	Young’s Modulus (GPa)		Poisson’s Ratio		Volume Fraction of SiC Particle (vol%)
	Fitting Results	Simulation Results	Fitting Results	Simulation Results	
1	445	445	0.355	0.354	67.2
2	390	391	0.360	0.360	44.1
3	348	347	0.367	0.365	23.8
4	325	322	0.371	0.374	11.5

**Table 3:** Comparison of fitting and simulation results of thermal properties of optimized transition layers.

Layers	$\mathrm{Thermal}\;\mathrm{Conductivity}\;(\frac{W}{m\cdot K})$		$\mathrm{Heat}\;\mathrm{Capacity}\;(\frac{J}{kg\cdot K})$		$\mathrm{Coefficient}\;\mathrm{of}\;\mathrm{Thermal}\;\mathrm{Expansion}\;(10^{-6}/K)$	
	Fitting Results	Simulation Results	Fitting Results	Simulation Results	Fitting Results	Simulation Results
1	51.73	51.81	289.2	288.9	5.68	5.66
2	60.41	60.49	271.1	270.8	6.09	6.12
3	68.90	69.03	257.2	256.5	6.49	6.54
4	74.47	74.71	249.6	250.3	6.75	6.88

## Data Availability

The data presented in this study is available on request from the corresponding author. The data are not publicly available due to privacy restrictions.
